# Direct Growth of a Polypyrrole Aerogel on Hollow CuS Hierarchical Microspheres Yields Particles with Excellent Electromagnetic Wave Properties

**DOI:** 10.3390/polym10111286

**Published:** 2018-11-19

**Authors:** Zhi Zhang, Xuliang Lv, Guangzhen Cui, Mingxu Sui, Xiaodong Sun, Songlin Yu

**Affiliations:** 1 Key Laboratory of Science and Technology on Electromagnetic Environmental Effects and Electro-Optical Engineering, The Army Engineering University, Nanjing 210007, China; zhangnjjn@163.com (Z.Z.); xllu1957@126.com (X.L.); cgzovexyh@163.com (G.C.); plasmx@126.com (M.S.); 2 Research Institute for National Defense Engineering of Academy of Military Science PLA China, Beijing 100036, China

**Keywords:** polypyrrole, core–shell, electromagnetic absorption, dielectric loss

## Abstract

A current hot topic in polymer science is the development of electromagnetic wave-absorbing materials with desired properties (i.e., proper impedance matching and strong attenuation capability), but it presents a considerable challenge. In this work, solvothermal, and self-assembled polymerization were employed for the controlled fabrication of a uniform polypyrrole (PPy) aerogel coated on hollow CuS hierarchical microspheres (CuS@PPy). The PPy coating thickness of the heterostructure could be tuned by varying the feeding weight ratios of CuS/pyrrole monomer. The electromagnetic wave absorption properties of the CuS@PPy composites were estimated to be in the frequency range 2–18 GHz. The as-prepared Sample B (fabricated by the addition of 35 mg CuS) showed a maximum reflection loss (RL) of −52.85 dB at a thickness of 2.5 mm. Moreover, an ultra-wide effective bandwidth (RL ≤ −10 dB) from 9.78 to 17.80 GHz (8.02 GHz) was achieved. Analysis of the electromagnetic properties demonstrated that the CuS@PPy had a remarkable enhancement compared to pure CuS platelet-based spheres and pure PPy, which can be attributed to the increased relatively complex permittivity and the promoted dielectric loss by the intense interfacial dielectric polarizations. We believe that the as-fabricated CuS@PPy can be a good reference for the fabrication of lightweight and optimal broadband absorbers.

## 1. Introduction

In recent years, reported information leaks as well as physiological damage to human bodies have brought considerable attention to electromagnetic irradiation [1,2,3,4,5,6]. Electromagnetic absorbers are a kind of functional material that can eliminate electromagnetic waves (EMWs) by converting their energy into thermal energy [7,8,9]. In general, the accepted requirements always include strong absorbing intensity, wide bandwidth, lightweight, and low cost [10]. In this regard, extensive studies have been done to develop excellent absorbers. Traditional ferromagnetic metals, including Fe_3_O_4_ [11,12], Co [13,14], NiO [15], and Fe [16], have been demonstrated to have promising attenuation capabilities. However, these materials are susceptible to corrosion, and have high density as well as insufficient absorption bandwidth [17]. To date, carbon-based materials such as carbon fibers (CFs) [18,19], carbon nanotubes (CNTs) [20,21], porous carbon spheres [22], and graphene or reduced graphene oxide (RGO) [23,24,25] have been selected as promising absorber candidates due to their high dielectric loss and low density [4]. However, the high conductivity of pure carbon materials always induce an eddy current effect as well as reflection. As a matter of fact, it is difficult for any one component to satisfy the requirements of ideal absorbers because of poor impedance matching, and the technology is not suitable for application if the electromagnetic wave cannot penetrate into the material.

According to the impedance matching condition, there is no electromagnetic wave reflecting on the surface of the absorber when |*Z*_in_/*Z*_0_| = 1 (*Z*_in_ is the input characteristic impedance of the absorber, *Z*_0_ = 376.7 Ω is the intrinsic impedance of free space.), and making the relative complex permittivity close to the relative complex permeability favors reducing the impedance mismatch between materials and free space [26]. Therefore, fabricating hybrids with different components to simultaneously obtain the proper impedance matching renders them eligible candidates as high-performance absorbers. For example, Wang et al. prepared cobalt/polypyrrole (Co/PPy) nanocomposites via in situ oxidation polymerization of pyrrole in an aqueous dispersion of Co nanoparticles (NPs) [27]. With a relative low filler loading, the minimum reflection loss (RL) value was around −33 dB. The effective absorption bandwidth (RL < −10 dB) for Co/PPy was 4.77 GHz (11.7–16.47 GHz) with a thickness of 2 mm. Quan et al. fabricated MoS_2_/rGO composites via a facile and effective hydrothermal approach [28]. The minimum RL was −67.1 dB at 14.8 GHz and the effective absorption bandwidth was 5.92 GHz (12.08–18.00 GHz) with a small thickness of 1.95 mm. Furthermore, structure modification is also an effective way to promote the electromagnetic absorption (EA) performance, such as core–shell structure, porous structure, hollow structure, etc. These unique structures can induce multiple reflection and scattering, which is favorable to improve the EA properties. Zheng et al. prepared Fe_3_O_4_@SiO_2_ core–shell nanostructures with different shell thicknesses and core diameter via a modified StÖber method [29], and the exhibited enhanced absorption properties gave forceful evidence that the as-prepared Fe_3_O_4_@SiO_2_ are attractive materials for EA applications. Zhang et al. synthesized a novel FeCo NPs-embedded nanoporous carbon composite (Fe–Co/NPC) [4]. Owing to the proper impedance matching and the multiple scattering induced by the porous structure, the minimum RL value and effective absorption bandwidth of the Fe–Co/NPC were far greater than those of commercial carbonyl iron powder under a very low thickness (1–1.5 mm).

Copper sulfide (CuS) is known as an important semiconductor [30,31,32]. More recently, the electromagnetic absorption performances of CuS materials and their hybrids have also been confirmed by some group studies [33,34]. Although the EA properties of CuS materials have made significant progress, they can hardly be satisfied by a low filler loading, which limits their further application. Permittivity plays crucial roles in controlling the proper impedance matching and the attenuation capabilities, and previous work gives us a great deal of inspiration in the use of permittivity regulation as a strategy to fabricate high-performance absorbers [35]. Polypyrrole (PPy) is a type of the intrinsically conducting polymers which has been investigated as a permittivity regulation for EA materials mainly result from its high dielectric property. Moreover, the lightweight of PPy aerogel after drying out which also matches the criteria of high-performance absorbers. In this work, CuS microspheres and PPy were successfully fabricated into a hierarchical core–shell structure. In the previous research presented by Peng et al. [36], CuS@PPy composite was developed to enhance the pseudo capacitive. The CuS microspheres used in this experiment and the polymerization process were completely different. To the best knowledge of us, this work reports for the first time that CuS@PPy microspheres are a promising material for EA application. Vector network analyzer (VNA) is an instrument for directly measuring the scattering parameters of microwave components. The scattering parameters characterize the direct relationship between the incident wave and the outgoing wave. The permittivity and permeability are calculated from the scattering parameters based on the standard Nicolson–Ross–Weir (NRW) algorithm [7]. The dielectric constant parameters as tested by the VNA were significantly improved by the coated PPy shells. The as-prepared Sample B exhibited outstanding EA performance with an extremely low filler loading (15 wt %). The minimum RL value reached −52.86 dB at a thickness of 2.5 mm, and the broadest effective bandwidth was achieved from 9.78 to 17.80 GHz (8.02 GHz). It was also found that the hierarchical structure and PPy shells aided in enhancing the attenuation ability by improving the impedance matching and the induced intensified interfacial polarization. The results show that CuS@PPy microspheres have an impressive EA capacity. 

## 2. Materials and Methods 

### 2.1. Materials

All chemicals were used directly without any further purification. Pyrrole (Py) monomer, copper sulfate pentahydrate (CuSO_4_·5H_2_O) and cetyltrimethyl ammonium bromide (CTAB) were purchased from GENERAL-REAGENT, Titan Scientific Co., Ltd., Shanghai, China. Sulfur powder (S), ferric chloride hexahydrate (FeCl_3_·6H_2_O), and ethylene glycol (EG) were purchased from Shanghai Sinopharm Chemical reagent Co. Ltd., Shanghai, China. Deionized water was obtained from Direct-Q3 UV, Millipore (Burlington, MA, USA).

### 2.2. Synthesis of Hollow Hierarchical CuS Microspheres

Hollow hierarchical CuS microspheres were prepared via a facile solvothermal method as reported previously [34]. In a typical procedure, 0.001 mol CuSO_4_·5H_2_O was first well-dispersed in EG (30 mL) under sonication for 0.5 h. Then, 0.001 mol CTAB which was well-dispersed in EG (30) was stepwise injected into the solution. After vigorous magnetic stirring for 0.5 h, 0.002 mol sulfur powder was introduced to the above mixture and kept stirring for 0.5 h. Then, the mixture was transferred into a Teflon-lined stainless-steel autoclave (100 mL capacity). Subsequently, upon sealing, the autoclave was maintained at 160 °C for 15 h. After cooling down to room temperature, the precipitate was centrifuged and rinsed with deionized water and absolute ethanol and dried in a vacuum oven at 40 °C for 12 h.

### 2.3. Synthesis of Core–Shell Structured CuS@PPy Microspheres

Briefly, a certain amount of as-prepared CuS microspheres and 0.33 g Py monomer were well-dispersed in 100 mL ethanol under magnetic stirring for 0.5 h, denoted as the solution A. Then, 3.1 g FeCl_3_·6H_2_O was dissolved in 100 mL ethanol via continuous magnetic stirring for 0.5 h (denoted as the solution B). Subsequently, the solution B was added dropwise to the solution B with rapid stirring. The resulting solution became black and was kept stirring for 20 h. The precipitate was filtered, washed, and dialyzed several times with deionized water and ethanol to remove impurities, and then dried at 40 °C for 12 h. For convenience, the CuS@PPy microspheres synthesized with different amounts of CuS microspheres (i.e., 25, 35, and 45 mg), were denoted as Samples A, B, and C, respectively.

### 2.4. Characterization and Measurement

The crystal structures of the as-prepared samples were analyzed with an X-ray diffractometer (XRD, D8-Advance, Bruker, Germany). The morphologies of samples were characterized by field emission scanning electron microscopy (FE-SEM, Hitachi S-4800, 3 kV) and high-resolution transmission electron microscopy (TEM, JEOL JEM-2100F). X-ray photoelectron spectra (XPS) were recorded using an ESCALAB 250Xi X-ray photoelectron spectrometer (Thermo Fisher Scientific, Waltham, MA, USA).

The relative complex permittivity (*ε*_r_) and permeability (*μ*_r_) were measured by a vector network analyzer (VNA, N5242A PNA-X, Agilent, Agilent Technologies Inc., Santa Clara, CA, USA) in the 2–18 GHz range using the coaxial measurement method. The mixture was then pressed into toroidal samples with an outer diameter of 7.00 mm and inner diameter of 3.04 mm. According to the transmission line theory, the theoretical RL of the heterostructures with different thicknesses can be calculated using the relative complex permittivity and permeability at a given frequency by the following equations [37,38,39,40]:

(1) $$\mathrm{RL}=20\;\log|\left(Z_{\mathrm{in}}-Z_{0}\right)/\left(Z_{\mathrm{in}}+Z_{0}\right)|,$$

(2) $$Z_{\mathrm{in}}=Z_{0}\sqrt{\frac{\mu_{r}}{\varepsilon_{r}}}\tan h\left(j\frac{2\pi fd\sqrt{\mu_{r}\varepsilon_{r}}}{c}\right),$$

where is *Z*_in_ is the input characteristic impedance of the absorber, *c* is the velocity of light in vacuum in m/s, *f* is the electromagnetic wave frequency in Hz, *d* is the thickness in meter (m), *Z*_0_ = 376.7 Ω is the intrinsic impedance of free space, *μ*_r_ is the complex permeability, *μ*_r_ = *μ*′ − *jμ*″, and *ε*_r_ is the complex permittivity, *ε*_r_ = *ε*′ − *jε*″.

## 3. Results

### 3.1. Sample Characterization

**Figure 1:**
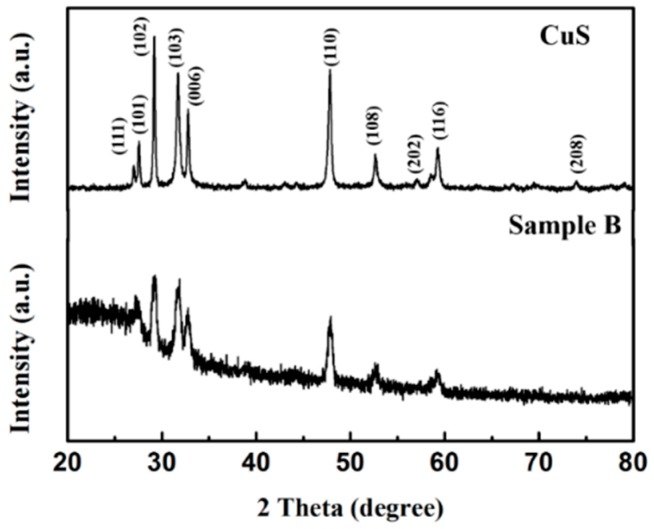
XRD patterns of pristine CuS and Sample B.

**Figure 2:**
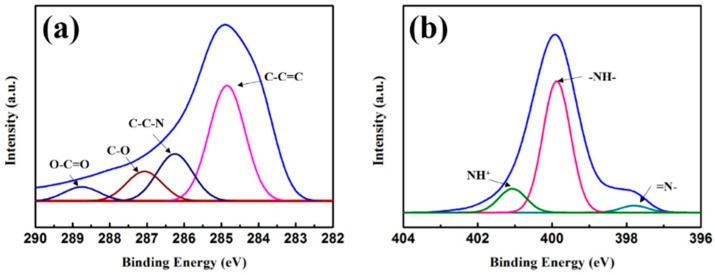
X-ray photoelectron spectroscopy (XPS) spectrum of (**a**) C 1s and (**b**) N 1s.

The crystal structures of the synthesized samples were investigated by XRD, and the corresponding patterns are shown in Figure 1 (pristine CuS and Sample B) and Figure S1 (Sample A and C). For the pristine CuS, the diffraction peaks were consistent with the values from the standard card of JCPDS Card No: 0464. Because of the high purity and crystallinity of the samples, no additional peak could be observed. PPy showed an amorphous structure as indicated by the broad XRD pattern around 20–30°. Note that the intensity of CuS diffraction peaks became weak with increasing PPy aerogel coating because of the increasing coating covering the CuS surface on the shell of the microspheres, and also due to the high amorphous nature. The chemical compositions were also characterized by XPS, and the results are shown in Figure 2, indicating the presence of C and N elements. It is well-known that XPS is a surface-sensitive quantitative spectroscopic technique. Its penetration depth is less than 10 nm, so no Cu or S elements could be detected. From this respect, it could also be proved that the CuS microspheres were uniformly coated by the PPy aerogel.

**Figure 3:**
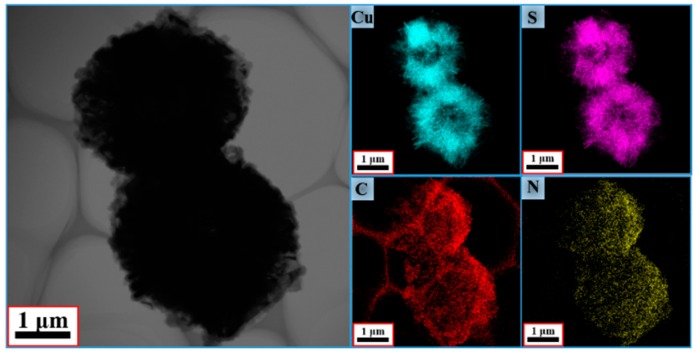
Elemental mapping images of Cu, S, C, and N.

**Figure 4:**
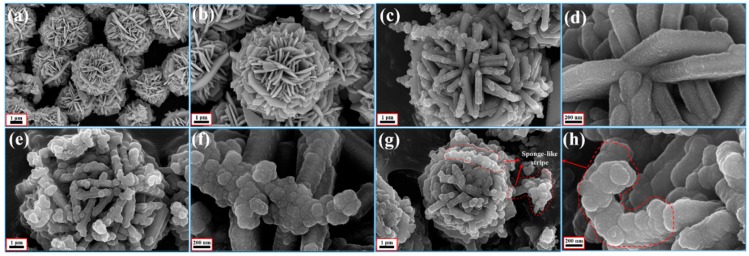
Different magnification SEM images of (**a**,**b**) hollow CuS, (**c**,**d**) Sample A, (**e**,**f**) Sample B, and (**g**,**h**) Sample C.

**Figure 5:**
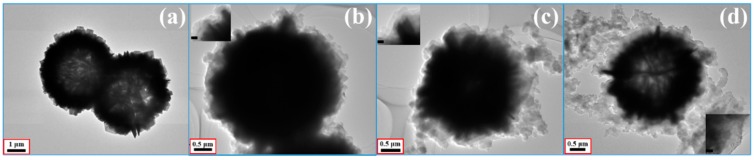
TEM images of (**a**) hollow CuS microspheres, (**b**) Sample A, (**c**) Sample B, and (**d**) Sample C. The insets of (**b**–**d**) are high-magnification TEM images of Samples A, B, and C, respectively. The inset black scale bars are 100 nm.

In Figure 3, the energy dispersive X-ray spectroscopy (EDS) elemental maps of Cu, S, C, and N demonstrate that the CuS microspheres (Cu and S elements) were equally coated by the PPy aerogel and maintained a consistent hollow and hierarchal morphology, indicating a perfect core–shell configuration. In this study, SEM and TEM were also used to characterize the morphologies and the microstructure. The starting hollow CuS microspheres are displayed in Figure 4a,b. The average diameter of these microspheres was about 4 μm, and the hierarchically structured pristine CuS was composed of thin disordered nanoflakes with an average thickness of about 15 nm. It is believed that this unique structure could provide more specific surface area for generating the bond with PPy aerogel. In addition, from the low magnification (scale bar: 1 μm) image, it can be seen that some partially broken microspheres clearly showed a hollow interior. CuS@PPy prepared in different experimental conditions are also shown in Figure 4. In the previous reported CuS/PPy, it was difficult to recognize the structure of the composite because of the heavy polymer coating. As revealed by SEM images (Figure 4c–h), the obtained three samples retained the original flower-like shape. The integrity of the hierarchical structure was well maintained after the gentle polymerization process. By comparing the following SEM images of the CuS@PPy samples, it can be found that the thickness of the flakes was increased by increasing the amount of the CuS starting component. In Figure 4g,h, some sponge-like stripes were generated by the excess of PPy particles. This kind of hierarchical hollow structure is beneficial for promoting the EA performance, which can induce more scatter and reflection [41,42]. We obtained TEM images for further characterization. The TEM image of CuS is shown in Figure 5a. The contrast between the light area in the center and the black area at the fringe further confirmed the hollow structures, and it could also be detected that the PPy shell thickness increased obviously from Figure 5b–d.

### 3.2. Electromagnetic Absorption Property

**Figure 6:**
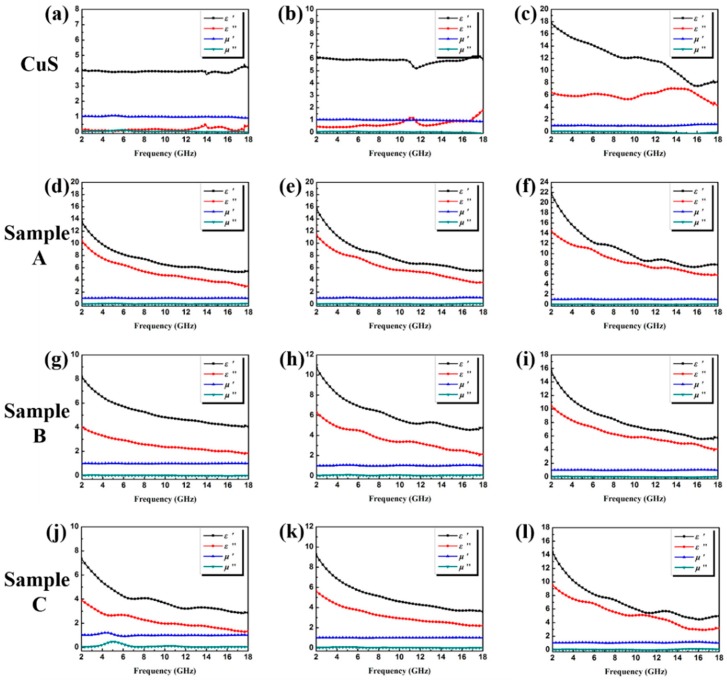
Frequency dependence of real and imaginary parts of complex permittivity and permeability of CuS with the filler loading of (**a**) 15 wt %, (**b**) 20 wt %, and (**c**) 30 wt %; Sample A with the filler loading of (**d**) 10 wt %, (**e**) 15 wt %, and (**f**) 20 wt %; Sample B with the filler loading of (**g**) 10 wt %, (**h**) 15 wt %, and (**i**) 20 wt %; Sample C with the filler loading of (**j**) 10 wt %, (**k**) 15 wt %, and (**l**) 20 wt %.

Herein, the coaxial line method is performed primarily to measure the complex relative permeability (*μ*_r_ = *μ*′ − *jμ*″) and permittivity (*ε*_r_ = *ε*′ − *jε*″) of the samples in the frequency range of 2–18 GHz, and the results are shown in Figure 6. Generally, the values of *ε*′ and *μ*′ represent the energy storage and inner dissipation capability of the incident electromagnetic wave, respectively [24,34,43]. The high values of *ε*′ and *ε*″ also indicate high storage capability and dielectric loss. In this study, owing to the absence of magnetic constituents in the composites, the values of *μ*′ and *μ*″ were all close to 1.0 and 0.0 [44]. All of the samples displayed in Figure 6 presented typical frequency-dependent permittivity. The values of ε′ and ε′′ decreased when the frequency increased in 2–18 GHz, which agrees with other reports. The increment can be attributed to the increase in the dipolar polarization and electrical conductivity due to the increasing loading ratio of the dielectric samples. Under the same filler loading ratio, the CuS@PPy samples showed a significant increase in comparison with the pristine CuS microspheres. All three of these samples displayed much larger *ε*′ and *ε*″ values, indicating high efficiency in storing and dissipating the electrical energy [45].

**Figure 7:**
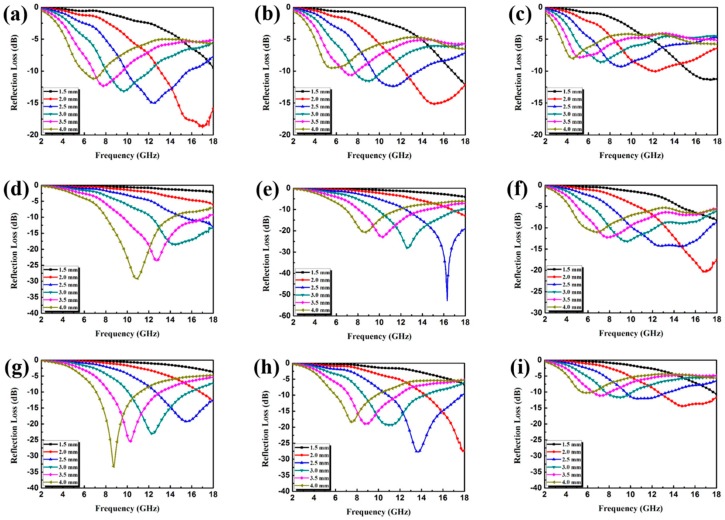
Reflection loss curves of paraffin composites containing (**a**) 15 wt %, (**b**) 20 wt %, and (**c**) 30 wt % Sample A; (**d**) 15 wt %, (**e**) 20 wt %, and (**f**) 30 wt % Sample B; (**g**) 15 wt %, (**h**) 20 wt %, and (**i**) 30 wt % Sample C.

**Figure 8:**
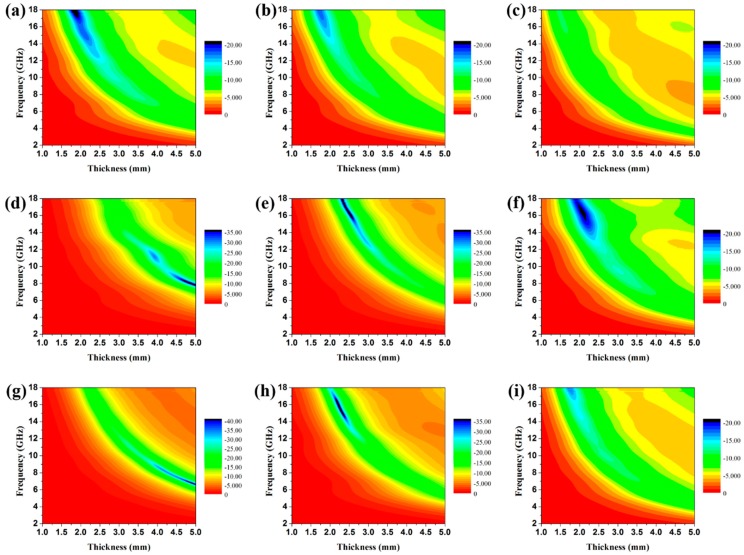
Contour maps of reflection loss of the paraffin composites containing (**a**) 15 wt %, (**b**) 20 wt %, and (**c**) 30 wt % Sample A; 15 wt % (**d**), 20 wt % (**e**) and 30 wt % (**f**) Sample B; (**g**) 15 wt %, (**h**) 20 wt %, and (**i**) 30 wt % Sample C.

The calculated RL curves of the pristine CuS microspheres and the various CuS@PPy samples with different thicknesses as well as different filler loading ratios are shown in Figure S2 and Figure 7. In addition, the corresponding contour maps of RL of the CuS@PPy samples are displayed in Figure 8. The optimal RL peaks all shifted toward a lower frequency with increasing thickness. This phenomenon can be interpreted from the fact that the formation of quarter-wavelength attenuation requires the absorbing thickness to meet the phase matching conditions, which can be recorded as *t*_m_ = *nc*/(4*f*_m_$\sqrt{\left|\varepsilon_{r}\right|\left|\mu_{r}\right|}$) (*n* = 1, 3, 5, …) [28], where *c* is the velocity of light in vacuum in m/s, *ε*_r_ = *ε*′ − *jε*″. *t*_m_ represents the matching thickness in m while *f*_m_ represents the peak frequency in Hz. In Figure S2, it can be seen that the pristine CuS exhibited poor RL characteristics, hardly less than −10 dB in the thickness range of 1.5–4.0 mm. When the ratio increased to 30 wt %, the minimum RL value reached −20.75 dB, but the effective bandwidth is merely 2.36 GHz, which hardly meets the requirements of an ideal absorber. This is because the relatively low dielectric constant results in poor impedance matching and weak attenuation capability. In view of Figure 7, it can be concluded that the EA performance of CuS@PPy samples was substantially enhanced relative to the pristine CuS microspheres. For Sample A, the best EA performance was achieved when the filler loading ratio was 15 wt %. From the dielectric parameters shown in Figure 6, it should be noted that the dielectric constants were too high with the increasing filler loading ratio—the higher concentration in these materials led to opposite EA properties. Such high permittivity could result in poor impedance matching, and the electromagnetic waves were reflected at the surface rather than penetrating into the absorbing materials. In Figure 7d, Sample B with a loading of only 10 wt % even showed a relatively strong attenuation capability. The minimum RL value was around −30 dB with the thickness of 4.0 mm, and the corresponding effective bandwidth was 6.01 GHz. The efficient frequency bandwidth is essential for practical application, and such a broad effective bandwidth makes this material more competitive. When the filler loading ratio reached 15 wt %, the minimum RL reached −52.86 dB with the thickness of 2.5 mm, and the broadest effective bandwidth was achieved when the thickness was 3.0 mm. A corresponding effective bandwidth from 9.78 to 17.80 GHz (8.02 GHz) was obtained. Such a broad bandwidth is better than is seen in numerous existing electromagnetic absorbers which have been reported previously. With the increasing filler loading ratio, the complex permittivity increased while the characteristic of impedance matching decreased, thus resulting in the deterioration of the EA performance. It can be concluded that the enhanced EA performance can be ascribed to the intensified polarization of the CuS@PPy core–shell interface and that a proper PPy shell thickness is also essential for maximizing the interface effect.

**Figure 9:**
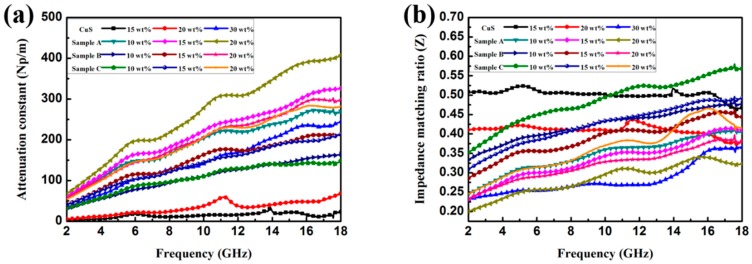
Frequency dependence of (**a**) attenuation constant ratio and (**b**) impedance matching for paraffin composites containing different loading ratios of CuS and CuS@PPy (polypyrrole) samples.

One important constant determining the attenuation properties of the absorbers is the attenuation constant (α), which can be determined as follows [37,46]:

(3) $$\alpha =\frac{\sqrt{2}\pi f}{c}\times \sqrt{\left(\mu^{\prime \prime }\varepsilon^{\prime \prime }-\mu^{\prime }\varepsilon^{\prime }\right)+\sqrt{{\left(\mu^{\prime \prime }\varepsilon^{\prime \prime }-\mu^{\prime }\varepsilon^{\prime }\right)}^{2}+{\left(\mu^{\prime }\varepsilon^{\prime \prime }+\mu^{\prime \prime }\varepsilon^{\prime }\right)}^{2}}},$$

where c is the velocity of light in a vacuum. The impedance matching ratio *Z* (|*Z*_in_/*Z*_0_|) is another important factor for judging the EA properties [4]. The detailed attenuation constants as well as the *Z* values of theses samples are displayed in Figure 9. From the results, it can be concluded that a high imaginary permittivity will cause the high value of *α*. The attenuation abilities of the core–shell-structured CuS@PPy samples were relatively higher than the pristine CuS in same filler loading ratio because of the elevated imaginary permittivity. In Figure 9b, pristine CuS (15 wt %) had the optimal impedance matching ratio because of the lowest complex permittivity [47]. On the contrary, the worst impedance matching ratio and the highest *α* value of Sample A (20 wt %) mainly resulted from its comparatively highest complex permittivity, which can be proved by the analysis in Figure 6. Attenuation capability and impedance matching are two key factors which should be considered to realize optimal EA materials [48]. Therefore, Sample B and Sample C, which had moderate attenuation constant values and impedance matching ratios can be deduced as qualified electromagnetic absorbers.

## 4. Conclusions

To summarize, we constructed uniform PPy aerogels coated on hollow CuS hierarchical microspheres (CuS@PPy) with controllable shell thicknesses via solvothermal and self-assembled polymerization. The as-prepared core–shell-structured CuS@PPy samples showed remarkable enhancement in EA properties. As the CuS contents and filler loading ratio vary, Sample B exhibited the best EA performance. When the filler loading ratio reached 15 wt %, the minimum RL reached −52.86 dB with the thickness of 2.5 mm, and the broadest effective bandwidth was achieved when the thickness is 3.0 mm. Attenuation constant and impedance matching ratio analysis also proved that Sample B (15 wt %) had the optimal compatibility. The excellent EA properties are attributed to the moderate impedance matching and strong dielectric loss. Moreover, the multiple reflection and scatter induced by the unique hierarchal core–shell structure are also beneficial to the attenuation. As a result, the obtained CuS@PPy samples with high absorption intensity and broad bandwidth can be expected to be qualified absorbers.

## Supplementary Materials

The following are available online at http://www.mdpi.com/2073-4360/10/11/1286/s1. Figure S1: XRD patterns of (a) Sample A and (b) Sample B; Figure S2: Reflection loss curves of paraffin composites containing 15 wt % 20 wt % and 30 wt % CuS under the thickness of 2.5 mm.
