# Supplementary material for: Direct Growth of a Polypyrrole Aerogel on Hollow CuS Hierarchical Microspheres Yields Particles with Excellent Electromagnetic Wave Properties
Source: Polymers (Basel). 2018 Nov 19;10(11):1286. doi: 10.3390/polym10111286 (PMC6401766; doi:10.3390/polym10111286)
Supplement: Supplementary file 1 [file polymers-10-01286-s001.pdf]

polymers
MDPI

Article

Direct growth of Polypyrrole aerogel on Hollow CuS hierarchical microspheres towards Excellent and Lightweight Electromagnetic Wave absorber

Zhi Zhang [1], Xuliang Lv [1], Guangzhen Cui [1], Mingxu Sui [1], Songlin Yu [2,*] and Xiaodong Sun [1,*]

[1] Key Laboratory of Science and Technology on Electromagnetic Environmental Effects and Electro-optical Engineering, The Army Engineering University, Nanjing, 210007, PR China;
zhang9511@hotmail.com (Z.Z.); xllu1957@126.com (X.L.); cgzjmz@hotmail.com (C.G.); plasmx@126.com (M.S.)

[2] Research Institute for National Defense Engineering of Academy of Military Science PLA china, Beijing, 100036, PR China;

* Correspondence: songlinyu@hotmail.com (Y.S.); xiaodongsun1001@hotmail.com (S.X.)

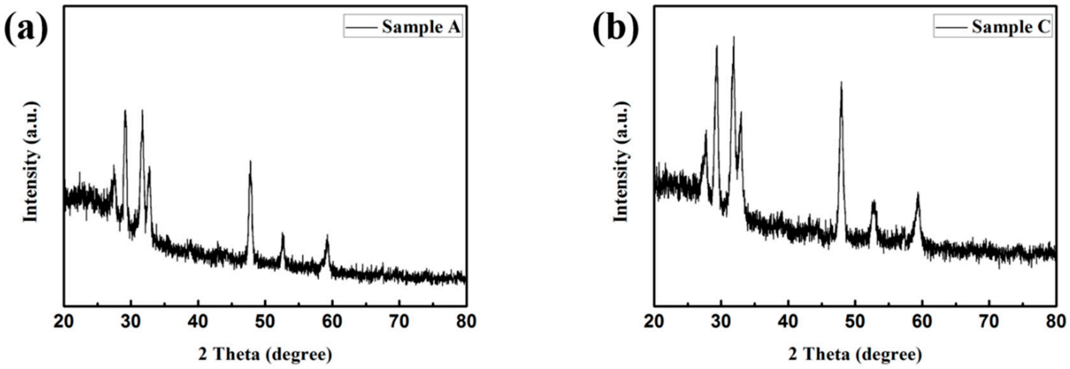

Figure S1. XRD patterns of Sample A (a) and Sample B (b).

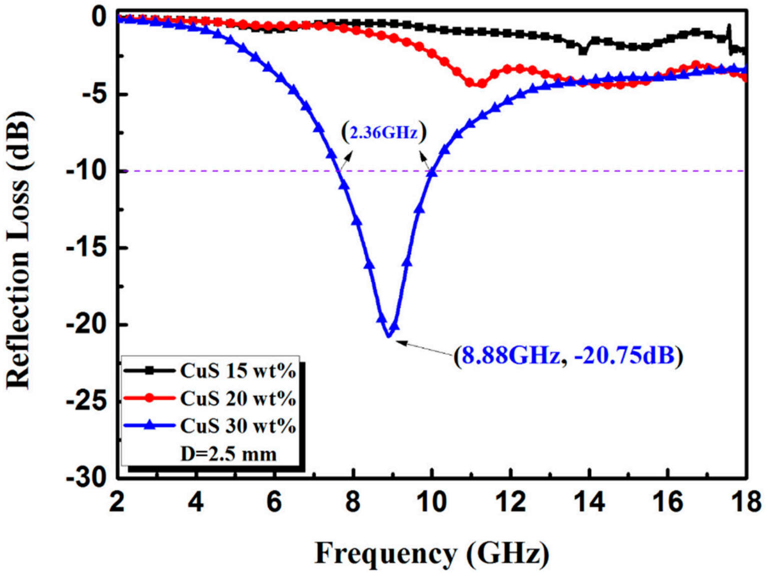

Figure S2. Reflection loss curves of paraffin composites containing 15 wt% 20 wt% and 30 wt% CuS under the thickness of 2.5 mm.
